# Comparing the Outcomes of Thrombus Aspiration Versus Standard Percutaneous Coronary Intervention in ST-Segment Elevation Myocardial Infarction Patients Undergoing Primary Percutaneous Coronary Intervention

**DOI:** 10.7759/cureus.82545

**Published:** 2025-04-18

**Authors:** Deepak Karumuri, Ramesh Sankaran, Sadhanadham S, Nagendra Boopathy Senguttuvan, Balasubramanian Jayanthy Venkata, Vinod Kumar Balakrishnan, Preetam Krishnamurthy, Vijay Muthyala

**Affiliations:** 1 Cardiology, Sri Ramachandra Institute of Higher Education and Research, Chennai, IND; 2 Interventional Cardiology, Sri Ramachandra Institute of Higher Education and Research, Chennai, IND; 3 Critical Care Medicine, Sri Ramachandra Institute of Higher Education and Research, Chennai, IND

**Keywords:** ejection fraction, outcome, percutanoeus coronary intervention, st elevation myocardial infarction (stemi), thrombus aspiration

## Abstract

Background

ST-segment elevation myocardial infarction (STEMI) is a major cause of cardiovascular mortality, mediated by coronary plaque rupture or erosion and acute thrombus formation. Early reperfusion is essential for reducing mortality and improving patient survival. Managing thrombus load during STEMI can be challenging and may require additional interventions beyond the conventional percutaneous coronary intervention (PCI) approach. This study aims to evaluate the impact of thrombus aspiration in STEMI patients undergoing primary PCI and its effect on procedural success and outcomes compared to standard PCI.

Materials and methods

This retrospective study was conducted at Sri Ramachandra Institute of Higher Education and Research (SRIHER), a university hospital in Chennai, India, by collecting baseline data from the medical records of 166 acute STEMI patients who underwent primary PCI. Patients were categorized into two groups based on whether they received thrombus aspiration or not. The primary outcomes studied were procedural success and in-hospital mortality, while secondary outcomes included safety, improvement in left ventricular function post-procedure, and the duration of hospital/ICU stay. The study was conducted in accordance with ethical guidelines and ensured patient confidentiality.

Results

A total of 166 patients were analyzed in this study, with chest pain being the major presenting symptom. Most patients had anterior wall myocardial infarction (AWMI) (75 (45.18%)), followed by inferior wall myocardial infarction (IWMI) (49 (29.52%)) and inferoposterior wall myocardial infarction (IPWMI) (29 (17.47%)). Most patients had a lesion in the left anterior descending artery (LAD), followed by the right coronary artery (RCA). The study found no significant difference in procedural success and outcomes between thrombus aspiration and conventional PCI, with both methods showing substantial improvements in ejection fraction on follow-up. Hospital and ICU stays were also not statistically different between the groups.

Conclusion

The study suggests that routine thrombus aspiration does not significantly improve myocardial recovery compared to conventional PCI, emphasizing the need for careful patient selection and optimal pharmacotherapy post-PCI.

## Introduction

In ST-segment elevation myocardial infarction (STEMI), early reperfusion decreases mortality. Pathogenesis involves the formation and progression of atherosclerosis, followed by the sudden erosion or rupture of atherosclerotic plaque, leading to thromboembolism that partially or completely obstructs the coronary artery lumen [1]. In patients with STEMI, the goal is to reestablish patency of the infarct-related artery (IRA) as soon as possible, restore adequate myocardial tissue perfusion, and preserve myocardium [2]. However, a large thrombus load in acute coronary syndrome is a significant challenge for interventional cardiologists to achieve myocardial perfusion, which can decrease the probability of procedural success [3,4,5]. A substantial thrombus burden at the lesion site may elevate the risk of distal embolisation and microvascular blockage, even after stent implantation or balloon dilatation [6,7]. Periprocedural complications include an increased risk of reperfusion injury, arrhythmias, contractile dysfunction, microvascular impairment, and permanent myocardial damage [8].

Thrombus aspiration was increasingly used in primary PCI to reduce thrombus burden. However, the benefits of the same are debated, as large studies have shown no benefit from the routine use of thrombus aspiration. Observational analyses indicate that manual thrombus aspiration is less successful for patients with prolonged ischemia periods, leading to the idea that thrombus aspiration is advantageous for patients who appear early [9]. Some contend that individuals with extended ischemia durations possess an organized thrombus, benefiting more from thrombus aspiration. Hence, our study aims to assess the effect of thrombus aspiration during primary PCI on mortality and procedural success (defined as achieving TIMI flow grade 3), procedure-related complications, ICU and hospital stay duration, morbidity, and mortality, in comparison with standard primary PCI without thrombus aspiration in STEMI patients.

## Materials and methods

Study design and population

Study Setting

This is a retrospective, single-centre study conducted at Sri Ramachandra Institute of Higher Education and Research (SRIHER), a university hospital in Chennai, India. Patient data were identified retrospectively from the HMIS database of SRIHER.

Inclusion Criteria

The study included patients aged 18 years or older with a confirmed diagnosis of acute STEMI, based on standard ECG criteria, and eligible for primary PCI according to current guidelines (e.g., ACC/AHA/SCAI). Patients were retrospectively categorized into either the thrombus aspiration group or the no thrombus aspiration group based on the intervention received. Thrombus aspiration was performed at the operator’s discretion. A total of 166 patients were enrolled in the present study.

Exclusion Criteria

STEMI patients who were successfully thrombolysed were excluded from the study. Additionally, patients with contraindications to PCI and bleeding risk, as well as pregnant and breastfeeding women, were excluded.

Sampling Technique

A convenience sampling method was used for data collection.

Study Location

The study was conducted at Sri Ramachandra Institute of Higher Education and Research (SRIHER), Chennai, India.

Study Duration

The study duration was one year, and STEMI patients undergoing primary PCI between November 2023 and November 2024 were enrolled.

Data collection and management

Baseline data were retrospectively collected from the medical records database. This included demographic information (age, sex, ethnicity), medical history (comorbidities, prior cardiovascular events, risk factors), laboratory results (e.g., cardiac enzymes, complete blood count), ECG findings, and echocardiographic data of left ventricular ejection fraction (LVEF). Angiogram videos were reviewed to assess lesion characteristics. Procedural data from the primary PCI, such as the type of stent(s) used, thrombolysis in myocardial infarction (TIMI) flow grade before and after thrombus aspiration (if applicable), final TIMI 3 flow after the procedure, and any procedural complications (e.g., dissection, perforation), were also collected.

Primary and secondary outcomes

The primary outcomes assessed were procedural success, defined as achieving TIMI flow grade 3 post-primary PCI, death, repeat target vessel revascularisation, and repeat hospitalisation. Secondary outcomes included the length of ICU and hospital stay, and post-procedure improvement in left ventricular (LV) function measured by 2D echocardiography at one-month follow-up.

Statistical analysis

Descriptive statistics were used to summarise patient characteristics, procedural details, and outcomes. Continuous variables were compared using t-tests or non-parametric tests as appropriate. Categorical variables were compared using chi-square tests or Fisher’s exact test. Statistical analysis was performed using MedCalc statistical software, and a p-value of <0.05 was considered statistically significant.

Ethical considerations

The study was conducted in accordance with the Declaration of Helsinki and Good Clinical Practice guidelines. The institutional ethics committee approved the research study (IEC details: REF: CSP-MED/24/JUL/106/224). Patient confidentiality was maintained throughout the study.

## Results

Our study enrolled 166 patients with a mean age of 56.06 ± 12.79 years. In the study population, 137 (82.53%) were male, and the remaining 29 (17.47%) were female. The notable comorbidities observed were Type II Diabetes Mellitus in 114 patients (68.67%), followed by dyslipidaemia in 92 patients (55.43%) and systemic hypertension in 69 patients (41.57%). Chest pain was the predominant presenting complaint, observed in 164 patients (98.79%). Anterior wall myocardial infarction (AWMI) was the most common type of myocardial infarction, seen in 75 patients (45.18%), followed by inferior wall myocardial infarction (IWMI) in 49 patients (29.52%) and inferoposterior wall myocardial infarction (IPWMI) in 29 patients (17.47%). Among the study population, 78 patients (46.99%) had a lesion on the left anterior descending artery (LAD), followed by the right coronary artery in 63 patients (37.95%). The average duration of hospital stay among patients in both groups was 3-4 days for 115 patients (69.28%), and 73 patients (43.97%) stayed in the intensive care unit for 2 days. Thrombus aspiration was performed in 57 patients (34.34%), while the remaining 109 patients (65.66%) received no thrombus aspiration. All patients received appropriate dual antiplatelet therapy post-procedure in both groups.

Comparison of thrombus aspiration and conventional PCI outcomes

The outcomes of thrombus aspiration and conventional PCI were compared, and the observations are displayed in Table 1. Mortality was noted in both groups, but the difference was not statistically significant (P = 0.8488). The pre-TIMI flow and improvement in TIMI flow across both groups did not show any statistically significant difference. Other study variables such as no-flow/slow-flow (P = 0.5164), repeat hospitalisation (P = 0.6902), and repeat target lesion revascularisation (TLR) (P = 0.8137) also did not show significant differences between the two methods.

**Table 1:** Comparison of thrombus aspiration and conventional PCI outcomes. PCI: Percutaneous Coronary Intervention; TLR: Target Lesion Revascularisation; TIMI: Thrombolysis In Myocardial Infarction; Pre-TIMI / Post-TIMI: Pre- or Post-Intervention Thrombolysis In Myocardial Infarction Flow Grade.

Variables	Thrombus Aspiration	Conventional PCI	Chi-square	P-value
No flow / Slow flow			0.421	0.5164
– Yes	4	19		
– No	53	90		
Repeat hospitalisation			0.159	0.6902
– Yes	9	15		
– No	48	94		
Repeat TLR			0.056	0.8137
– Yes	3	2		
– No	54	107		
Pre-TIMI flow			2.371	0.1236
– 0–1 flow	50	73		
– >1 flow	7	36		
Post-TIMI flow			0.018	0.8937
– Grade 3 flow	56	107		
Death			0.036	0.8488
– Yes	2	5		
– No	55	104		

Comparison of ejection fraction

The left ventricular ejection fraction (LVEF) was assessed pre- and post-procedure, and the outcomes are shown in Table 2. Overall, ejection fraction improved significantly in both groups (P < 0.0001). In the thrombus aspiration group, patients showed an increase in ejection fraction from 40.81 ± 6.65% to 46.28 ± 8.69%, which was statistically significant (P = 0.0075). Similarly, in the conventional PCI group (no thrombus aspiration), the ejection fraction improved from 43.40 ± 7.99% to 47.48 ± 9.81%, also statistically significant (P = 0.0014). However, the difference in improvement between the two groups was not statistically significant.

**Table 2:** Comparison of left ventricular ejection fraction.

	Overall				Thrombus Aspiration				Conventional Management			
	Pre-Procedure	Post-Procedure	t-statistic	P-value	Pre-Procedure	Post-Procedure	t-statistic	P-value	Pre-Procedure	Post-Procedure	t-statistic	P-value
LV Ejection Fraction (%)	42.54±7.61	47.08±9.39	4.546	<0.0001	40.81±6.65	46.28±8.69	2.977	0.0075	43.40±7.99	47.48±9.81	3.416	0.0014

Hospital and ICU stays were also evaluated, and the results are summarized in Table 3. The duration of both hospital and ICU stay did not differ significantly between the two groups.

**Table 3:** Comparison of hospital and ICU stay between groups. PCI: Percutaneous Coronary Intervention.

Variables	Thrombus Aspiration	Conventional PCI	t-statistic	P-value
Hospital stay (days)	3.85 ± 1.32	3.86 ± 1.51	0.0108	0.9914
ICU stay (days)	1.91 ± 1.30	1.80 ± 0.84	–0.673	0.5021

## Discussion

Our study did not show that the routine use of thrombus aspiration improved outcomes in terms of death, repeat hospitalisation, repeat target lesion revascularisation (TLR), or shortening ICU/hospital stay. The mean age of the study population was 56.06 ± 12.79 years, with a male preponderance of 137 (82.53%) noted, aligning with previous studies [10-12]. Type II Diabetes Mellitus (114; 68.67%), hypertension (69; 41.57%), and dyslipidaemia (92; 55.42%) were significant comorbidities among the study population, which are well-known risk factors for acute coronary syndrome [13-15].

The results of thrombus aspiration versus traditional PCI revealed no significant differences in the mean duration of hospital stay, which was 3-4 days, and ICU stay, which was two days [16]. Other critical measures, including no flow/slow flow (P = 0.5164), repeat hospitalisation (P = 0.6902), repeat TLR (P = 0.8137), and death (P = 0.8488), showed no significant differences between the two groups. Early trials, including Thrombus Aspiration during Primary Percutaneous Coronary Intervention Study (TAPAS), indicated improved outcomes with thrombus aspiration, showing a reduction in all-cause and cardiac mortality at one year [17]. However, the TOTAL study, similar to our study, determined that routine thrombus aspiration during primary PCI did not decrease cardiovascular mortality or recurrent myocardial infarction but marginally increased the risk of stroke [18]. A meta-analysis conducted by Jolly SS et al. on the use of thrombus aspiration in STEMI patients revealed no significant advantage for mortality or myocardial perfusion [19]. No notable variations in the improvement of TIMI flow post-procedure were observed between the two groups, suggesting that thrombus aspiration may not reliably enhance procedural success despite its proposed advantages in minimising distal embolisation [20]. According to the TOTAL and TASTE (Thrombus Aspiration in STEMI in Scandinavia Trial) studies, these findings support the conclusion that routine thrombus aspiration does not offer significant benefits in terms of mortality or procedural success (Table 4) [4, 18]. Both groups showed improved EF post-procedure, with no significant difference between the two groups [21]. Thrombus aspiration can improve coronary flow and myocardial salvage, thereby enhancing EF [22]. Early intervention, irrespective of thrombus aspiration in primary PCI, reduces infarct size and increases left ventricular recovery [23]. In selected patients with a significant thrombus burden, aspiration with newer devices that can retrieve larger volumes of thrombus may produce better outcomes. These findings warrant further studies with novel therapies. Additionally, studies targeting specific subgroups of patients, such as those with large infarcts or high thrombus burden, may yield more positive results and provide further insights.

**Table 4:** Major trials of thrombus aspiration. PCI: Percutaneous Coronary Intervention; STEMI: ST-Segment Elevation Myocardial Infarction; CABG: Coronary Artery Bypass Grafting; NYHA: New York Heart Association. Study data adapted from Vlaar PJ et al. [17], Svilaas T et al. [21], Ito H et al. [22].

Study	TAPAS [17]	TASTE [22]	TOTAL [21]
Study design	Randomised, single-centre, prospective	Randomised, multi-centre, prospective	Randomised, multi-centre, prospective
Inclusion and exclusion criteria	Patients underwent either thrombus aspiration or conventional treatment before initial coronary angiography. Exclusion criteria: rescue PCI after thrombolysis and known existence of a concomitant disease with a life expectancy of less than 6 months.	STEMI and ability to provide oral consent <24 hours of symptoms; correspondence between ECG and angiographic findings. Exclusion criteria: need for emergency coronary artery bypass grafting, age <18 years, previous randomisation in TASTE.	Patients presenting with symptoms of myocardial ischaemia lasting ≥30 minutes and definite ECG changes indicating STEMI, referred for primary PCI and randomised within 12 hours of symptom onset. Exclusion criteria: (1) Age ≤18 years, (2) Prior coronary artery bypass surgery (CABG), (3) Life expectancy <6 months due to non-cardiac conditions, (4) Fibrinolytic therapy for the qualifying STEMI event.
Primary outcome	Cardiac death or non-fatal reinfarction after 1 year; analysis by intention to treat	All-cause mortality at 30 days	Composite of death from cardiovascular causes, recurrent myocardial infarction, cardiogenic shock, or new or worsening NYHA class IV heart failure within 180 days
Secondary outcome		Time to rehospitalisation with reinfarction at 30 days; time to stent thrombosis at 30 days	Key secondary outcomes included the primary efficacy outcome plus stent thrombosis or target-vessel revascularisation within 180 days and cardiovascular death within 180 days

The non-thrombus aspiration group had better baseline TIMI flow pre-procedure and a higher mean ejection fraction pre-procedure, which could have influenced the outcomes and results of our study. Other limitations include a small sample size, the retrospective and non-randomised nature of the study, and the lack of long-term follow-up to assess the sustained impact of the interventions. Furthermore, patients in both groups were not matched, which adds to the limitations of our study.

## Conclusions

Our study aligns with findings from major trials and guidelines, indicating that routine thrombus aspiration does not offer significant advantages over conventional PCI in improving mortality or reducing the incidence of no-flow/slow-flow, repeat TLR, or repeat hospitalisation. Thrombus aspiration is still used in practice when operators encounter a large thrombus burden. However, in accordance with current guidelines, routine thrombus aspiration is not warranted. Considering the heavy thrombus burden in a substantial number of STEMI presentations, novel therapies and newer devices are warranted to effectively manage high thrombus burden.
